# Effects of laparoscopic gastric plication (LGP) in patients with type 2 diabetes, one year follow-up

**DOI:** 10.1186/s40200-015-0188-4

**Published:** 2015-07-17

**Authors:** Mohammad Talebpour, Atieh Talebpour, Gilda Barzin, Reza Shariat Moharari, Mohammad Reza Khajavi

**Affiliations:** Department of surgery, Laparoscopic Ward, Sina Hospital, Tehran University of Medical Sciences, Tehran, Iran; Department of Anesthesiology, Tehran University of Medical Sciences, Sina Hospital, Hassan Abad Square, Tehran, Zip code: 1136746911 Iran

**Keywords:** Laparoscopic gastric plication, Diabetes, Obesity, Bariatric surgery

## Abstract

**Background:**

Obesity is a major risk factor for the development of type 2 diabetes mellitus. Surgery is one of the most effective treatments for morbid obesity. In a prospective cohort study, we examined the effects of Laparoscopic Gastric Plication (LGP) as a new restrictive technique on remission of type 2 diabetes mellitus.

**Methods:**

During six years of study from June 2007 through December 2013, 62 patients who underwent bariatric surgery were recruited for our study to determine the effects of weight loss. Sixty patients with diabetes mellitus type 2 were selected for a one year follow up period. The amount of weight loss, Fasting Blood Sugar (FBS), changes in the lipid profile, HbA1c and blood pressure were assessed during this period. The primary outcomes were safety and the percentage of patients experiencing diabetes remission.

**Results:**

Sixty patients with the mean age of 39.7 ± 12.8 years, ranging from 18 to 62 years, were enrolled in the study for an average 12 months of follow up. The maximal weight loss of 57 kg was achieved at average after six months. FBS significantly decreased during this period, and after one year, remission of diabetes was achieved in 92 % of patients. In five patients, diabetes was controlled with decrease in taking oral medications.

**Conclusions:**

Laparoscopic Gastric Plication (LGP) resulted in significant and sustained weight loss with minimal physiologic changes in gastrointestinal tract and ameliorated blood glucose control of type 2 diabetes in morbid obese patients.

## Introduction

Obesity and diabetes have reached to epidemic ratios and are responsible for major health and economic burdens. Diabetes is a chronic disease with potentially fatal complications. Approximately 26 million Americans have diabetes, while an estimated seven million patients with diabetes are even unaware of their condition. Early and intensive treatment of type 2 diabetes improves health outcomes and quality of life [1]. In this regard, weight control may be regarded as the most important aspect of type 2 diabetes management which significantly reduces morbidity and mortality [2]. In fact, recent evidence indicates that improvement in the control of blood glucose is related to the amount of weight loss [3]. Unfortunately, the currently available lifestyle and pharmacological interventions are provided to help for only little to moderate amounts of weight loss. Nonetheless, patients with diabetes experience greater difficulty in losing weight than those without diabetes [4].

Recently, many studies compared the efficacy of bariatric surgery (laparoscopic Roux-en-Y gastric bypass or laparoscopic sleeve gastrectomy) with medical treatment among patients with uncontrolled type 2 diabetes [5, 6]. New research has found that bariatric surgery is effective in abating the symptoms of type 2 diabetes in the short term as well as causing remission of diabetes in the long term [7]. In fact, observational evidence suggests that bariatric surgery is associated with a 60 %–80 % diabetes remission rate among morbid obese persons [8]. Noteworthy, bariatric surgery can help in the management of obesity and diabetes at the same time, however, considering the invasive nature of such procedures, bariatric surgery is not regarded as a significant part of diabetes control guidelines [9]. Among these procedures, Laparoscopic Gastric Plication (LGP) is a new, less invasive and restrictive technique that reduces the gastric volume by plication of the greater curvature [10].

In this prospective cohort study, we sought to determine the effectiveness of LGP induced weight loss in controlling type 2 diabetes.

## Materials and methods

This prospective cohort study was conducted on 60 morbid obese patients with recently diagnosed type 2 diabetes (<3 years) who were candidates for LGP surgery This trial was done from June 2007 to December 2013 at Sina hospital by a specialist general surgeon, who had fellowship in advanced laparoscopic surgery. The technique of LGP was performed according to the standard method based on the last paper of the author for all patients [11]. The Institutional Review Board (IRB) of Tehran University of Medical Sciences reviewed and approved the study protocol.

All patients underwent thorough preoperative evaluations including history and physical examination, nutritional status and psychiatric status evaluations, and specialty consultations as indicated. Preoperative evaluations also included complete blood count, urinalysis, serum chemistries, levels of fasting lipids including total cholesterol and triglycerides, electrocardiogram and abdominal sonogram. Patients with a Body Mass Index (BMI) greater than 30 and documented type 2 diabetes within the previous three years who were able to understand the study protocol were enrolled in the study. Also, presence of symptoms related to the diabetes complications such as renal impairment or diabetic retinopathy were considered as the exclusion criteria. The cut off value for the diagnosis of diabetes was 120 mg/dl of Fasting Blood Sugar (FBS) or higher, and hemoglobin A1C (HbA1c) levels ≥ 7 %. Progress of study was reviewed by the bariatric surgery team every four to six weeks throughout the study. The primary endpoint was the rate of remission of diabetes after one year, defined as FBS < 100 mg/dl and HbA1c levels < 6.5 % without pharmacologic treatment for at least one year.

Data were presented as mean ± standard deviation (SD) for continuous variables. For statistical analysis, SPSS software (SPSS for Windows 18.0; SPSS Inc., Chicago, IL, USA) was used. Changes from baseline in the metabolic parameters were evaluated with Bonferroni-corrected-repeated-measures ANOVA and P values <0.05 were considered significant.

## Results

Among the 62 patients who were candidates for LGP, 60 were eligible. The mean age of patients was 39.7 ± 12.8 years, ranging from 18 to 62 years. Twenty two patients were male (37 %) and 38 were female (63 %). Preoperative data of patients are outlined in Table 1. We were in contact with all the patients during the one year follow up.

**Table 1:** Preoperative patient characteristics

Characteristics	Range	Mean ± SD
Age(yr)	18–62	39.7 ± 12.8
Weight (kg)	95–156	125 ± 14.1
BMI(kg/m^2)^	31–55	42.4 ± 5.47
HbA_1_c (%)	8–12	9.8 ± 0.5
FBS(mg/dl)impending to diabetes	108–135	118.22 ± 6.43
FBS(mg/dl) overt diabetes	136–295	180.33 ± 36.12
Total cholesterol(mg/dl)	196–327	226.0 ± 42.4
Triglycerides (mg/dl)	187–307	227.61 ± 8.4
Mean blood pressure (mmHg)	85–111	105.9 ± 6.8

Twenty five patients were impending to diabetes. Among patients with overt diabetes, 28 patients did not take any drug and the rest of them took oral diabetes drugs and insulin. According to postoperative results (Table 2), participants had an average maximal weight loss of 42 kg after three months. This trend of weight loss was decreased after nine months. FBS declined significantly six months after surgery, which was correlated with the trend of weight loss during this period. The mean HbA1c for the entire cohort decreased progressively during the study from 9.8 % to 6.5 %. Most of this change occurred within the first six months. Remission of diabetes was achieved in 92 % of patients. In patients who experienced remission, diabetes medications were discontinued 4–22 weeks after surgery. In five patients, diabetes was controlled with decreased usage of oral diabetes medications. The changes in lipid profile and hypertension were correlated with the trend of weight loss after surgery. We did not find any correlation between remission of diabetes and age, gender or the class of weight category according to BMI during the study (P > 0.05).

**Table 2:** Postoperative patient’s outcomes during one year

Characteristics	3 month	6 month	9 month	12 month	P value
Mean weight loss(kg)	42 ± 14.21	57.21 ± 16.62	61.12 ± 14.21	69.05 ± 13.21	0.001
Mean FBS(mg/dl)	105.81 ± 12.5	98.76 ± 8.35	95.36 ± 7.24	91.25 ± 8.12	0.0001
HbA_1_c(%)	8.1 ± 0.2	6.5 ± o.6	6.1 ± o.4	5.6 ± o.4	0.002
Total cholesterol(mg/dl)	190 ± 8.2	178 ± 5.2	168 ± 6.2	150 ± 2.1	0.001
Triglycerides (mg/dl)	187 ± 6.3	171 ± 6.3	140 ± 5.4	115 ± 6.5	0.001
Mean blood pressure (mmHg)	92.03 ± 1.1	85.02 ± 2.3	76.12 ± 2.1	75.5 ± 1.4	0.001

## Discussion

In this study, we showed that gastric plication promotes remission of type 2 diabetes among morbid obese patients. Complete diabetes remission was achieved in 92 % of patients, with improvement of blood glucose levels in the remaining 8 % of patients. The observed improvement in the control of blood glucose was accompanied by substantial improvements in hypertension and dyslipidemia.

Most people with type 2 diabetes experience problems in losing weight. Although medications are useful in improving blood sugar, their benefits are quite limited among obese and particularly morbid obese patients. Unfortunately losing weight is very difficult with going on a diet, exercise and drug therapy for most patients. Therefore, surgery is very effective for losing weight among diabetic patients. In fact, weight loss decreases insulin resistance and improves blood sugar levels. The results of the trial done by Carlson et al. show that bariatric surgery reduces the long-term incidence of type 2 diabetes by 78 % in obese patients. Among patients with impaired fasting glucose, bariatric surgery reduced the risk by 87 %, and diabetes type 2 did not develop in 10 out of 13 obese patients who underwent bariatric surgery [12]. In addition, there is substantial evidence that bariatric surgery provides additional quality-of-life improvements and reduces mortality in patients with type 2 diabetes. It is noteworthy that Improvement of insulin sensitivity by means of weight loss may not be enough to induce the remission of diabetes if there is advanced destruction of beta cells. Also, the diabetes remission rate is inversely related to the duration of diabetes at the time of bariatric surgery [13].

Several mechanisms have been proposed for the improvement of blood glucose control through weight loss. There seems to be improved gastrointestinal hormone level changes after surgery such as Glucagon-like Peptide-1(GLP-1) [14]. After bariatric surgery, rapid delivery of partially digested nutrients to the distal bowel up-regulates the secretions of gut hormones such as glucagon-like peptide-1 (GLP-1). The result of the increased gut hormone secretion is an enhanced glucose-dependent insulin secretion as well as a number of other changes that cause improved glucose tolerance [15]. In a non-obese diabetic rat model, surgical diversion of the proximal bowel caused rapid improvement in diabetes without reduction of food intake or change in weight. Duodenal exclusion does not play a significant role alone in postsurgical amelioration of diabetes type 2 and restoration of gut hormone profile in lean diabetic rodents [16]. In our study, greater curvature of stomach was inverted to lumen of stomach and there was no resection or change in the physiology of gastrointestinal tract; however, weight loss and remission of diabetes occurred in most patients. Nonetheless, many aspects regarding the applications of surgical treatment of type 2 diabetes are still questionable and unresolved.

This study has several limitations. First, the follow up period was one year and it would be better to follow the patients for a longer period. Additionally, identifying the gut hormone levels before and after bariatric surgery may be helpful.

## Conclusion

Laparoscopic Gastric Plication (LGP) with minimal physiologic changes in the gastrointestinal tract ameliorates overall blood glucose control of type 2 diabetes in morbid obese patients.

## Abbreviation

LGP: laparoscopic gastric plication 1
DM T2: diabetes mellitus type 2
